# Isolation of the Main Pathogens Causing Postharvest Disease in Fresh *Angelica sinensis* during Different Storage Stages and Impacts of Ozone Treatment on Disease Development and Mycotoxin Production

**DOI:** 10.3390/toxins15020154

**Published:** 2023-02-13

**Authors:** Jihui Xi, Dongyun Yang, Huali Xue, Zhiguang Liu, Yang Bi, Yuan Zhang, Xi Yang, Suqin Shang

**Affiliations:** 1 College of Science, Gansu Agricultural University, Lanzhou 730070, China; 2 College of Food Science and Engineering, Gansu Agricultural University, Lanzhou 730070, China; 3 College of Plant Protection, Gansu Agricultural University, Lanzhou 730070, China

**Keywords:** *Angelica sinensis*, postharvest disease, identification of mold, ozone, mycotoxin production

## Abstract

*Angelica sinensis*, a Chinese herbal medicine, is susceptible to molds during storage, reducing its quality, and even generating mycotoxins with toxic effects on human health. Fresh *A. sinensis* was harvested from Min County of Gansu Province in China and kept at room temperature. Naturally occurring symptoms were observed during different storage stages. Molds were isolated and identified from the diseased *A. sinensis* using morphological and molecular biology methods. The impact of ozone treatment on postharvest disease development and mycotoxin production was investigated. The results indicated that *A. sinensis* decay began on day 7 of storage and progressed thereafter. Nine mold species were isolated and characterized: day 7, two *Mucormycetes*; day 14, *Clonostachys rosea*; day 21, two *Penicillium* species and *Aspergillus versicolor*; day 28, *Alternaria alternata* and *Trichoderma atroviride*; and day 49, *Fusarium solani*. Ozone treatment markedly inhibited the development of postharvest disease and the mycotoxin production (such as, patulin, 15-acetyl-deoxynivalenol, and sterigmatocystin) in the rotten tissue of *A. sinensis* inoculated with the nine isolates.

## 1. Introduction

The root of *Angelica sinensis* (Oliv.), family Umbelliferae, is used as a traditional Chinese herb. The bioactive components are terpenoids, phthalides, sterols, alkaloids, aromatic compounds, essential oils, fatty acids, and polysaccharides [1]. *A. sinensis* is commonly used for treatment of gynecological diseases, and its active ingredients include: phthalides, which have bioactivities including antitumor, analgesic, and neuroprotective effects [2]; polysaccharide compounds, which have therapeutic effects on acute liver injury [3]; and volatile oils, which have protective effects against atherosclerosis [4].

Due to its specific climate (low temperature and high humidity), Gansu Province ranks first for *A. sinensis* production in China, generating approximately 50,000 t annually, accounting for 70–90% of total production [5]. With increased *A. sinensis* plantation area, diseases affecting *A. sinensis* are becoming more obvious and seriously restricting *A. sinensis* yield and quality. *A. sinensis* diseases have frequently reported been by researchers in recent years; for example, *A. sinensis* root rot caused by *Fusarium acuminatum* and *Clonostachys rosea* [6], anthracnose caused by *Colletotrichum dematium* [7], and *A. sinensis* leaf spot disease caused by *Fusarium avenaceum* [8]. However, previous studies have focused primarily on *A. sinensis* diseases during growth in the field (pre-harvest), with few investigations documenting postharvest disease and mycotoxin accumulation in freshly-harvested *A. sinensis* under different storage conditions.

Recent research has suggested that fresh herbal medicine has higher medicinal value than processed material. For example, in terms of antidiabetic activity, fresh ginseng is more valuable than processed products containing it [9], while the dopamine and norepinephrine contents of fresh *Herba Portulacae* are higher than those of the dried product [10]. Therefore, the demand for fresh Chinese herbs is increasing; however, unfortunately, storage technology for fresh Chinese herbs is comparatively underdeveloped, and postharvest disease during storage is a serious concern. Contamination with pathogenic molds causes losses of approximately 15–25% Chinese herbs annually [11]. Moreover, the *A. sinensis* harvest period is mainly focused in late autumn in Gansu, during which temperatures are low and humidity high, while herbal moisture content is relatively high and the plant materials are rich in polysaccharides, proteins, and other organic components conducive to pathogen growth. In addition, due to a lack of drying equipment, *A. sinensis* is mainly kept in farmers’ yards for around two months to dry naturally after harvesting, then transferred to dealers, and *A. sinensis* can easily be infected by pathogens during the drying process, given appropriate temperature, humidity, and nutritional composition conditions, eventually, leading to disease occurrence. Postharvest disease not only seriously affects *A. sinensis* quality, but also reduces its medicinal value, and can even lead to the accumulation of mycotoxins, posing a potential threat to human health.

Mycotoxins are secondary metabolites produced by plant pathogenic molds under favorable conditions, with carcinogenic, teratogenic, mutagenic, and toxic effects on the human liver, kidney, nervous system, endocrine system, and immune system [12]. The function of traditional Chinese herbs is to treat disease, and if they are contaminated with mycotoxins, instead of ameliorating disease symptoms, they can have serious consequences for human health. Therefore, it is particularly important to control the postharvest disease and mycotoxin accumulation of fresh *A. sinensis* during storage after harvest.

Ozone is a strong oxidizing agent and is considered to have positive effects on the disease control of fruits and vegetables during storage [13]. For instance, Liang et al. [14] showed that ozone treatment had a good fresh-keeping effect on tomatoes. De Santis et al. [15] observed a significant detrimental effect of ozone treatment on garlic decay. However, the control of postharvest disease and mycotoxins accumulation in Chinese medicinal materials by ozone treatment has not been fully documented.

In this study, we investigated the development of postharvest disease in freshly harvested *A. sinensis*, isolated molds primarily causing postharvest disease during different stages of storage, and characterized them using morphological and molecular biological techniques. Furthermore, we also analyzed the control effect of ozone on postharvest diseases and mycotoxin accumulation in fresh *A. sinensis* inoculated with the nine molds.

## 2. Results

### 2.1. Disease Development in Fresh A. sinensis during Different Storage Stages after Harvest

**Figure 1:**
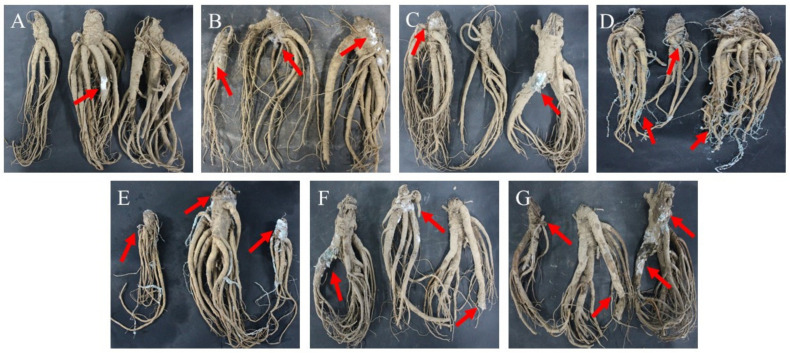
Naturally occurring symptoms in fresh *A. sinensis* during different stages of storage after harvest. (**A**) 7 d; (**B**) 14 d; (**C**) 21 d; (**D**) 28 d; (**E**) 35 d; (**F**) 49 d; (**G**) 63 d.

Disease symptoms of freshly-harvested *A. sinensis* became increasingly serious with extended storage time (Figure 1). After seven days of storage, slight symptoms of disease were observed and small amounts of hyphae were found on the surface of *A. sinensis*. Following storage for 14 days, white hyphae gradually diffused on the surface of the main root. After 21 days, green mycelia and spores appeared on the surface of *A. sinensis*; and after 28 days, mold colonies were expanded, mycelium growth was vigorous, fibrous roots were covered with green mycelia and spores. In *A. sinensis* stored for 35 days, colonies with complex species structures were observed, and some hyphae turned yellow and grey. After 49 days, multiple colonies were distributed over the surface of *A. sinensis* samples and the tissue was damaged. Further, storage for 63 days resulted in *A. sinensis* samples that were seriously diseased, with wrinkled, soft, and even rotten tissues.

### 2.2. Isolation of Molds Potentially Causing Postharvest Disease of A. sinensis during Different Storage Stages

A total of nine isolates with different morphological features were purified during the entire storage period and were named according to the time of isolation and purification, as follows: two isolates purified after 7 d of storage were named 7–1 and 7–2; a new isolate, 14–1, was purified at 14 d of storage; three new isolates were purified at 21 d of storage and termed 21–1, 21–2, and 21–3; two new isolates were purified at 28 d of storage, and named 28–1 and 28–2; and one new isolate was purified at 49 d of storage and named 49–1. No new isolates were purified at other storage stages.

### 2.3. Morphological Identification of Isolates

**Figure 2:**
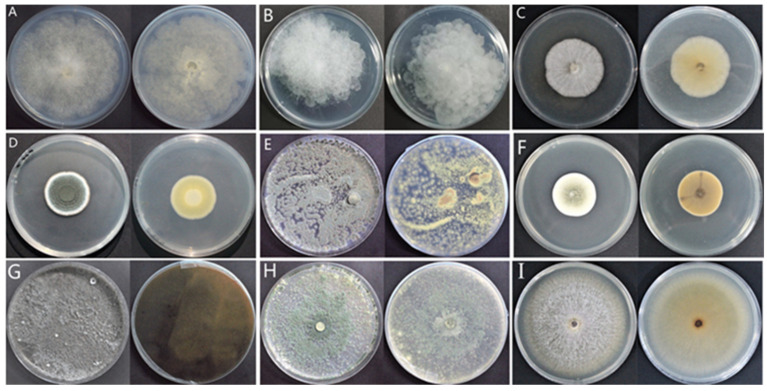
Colony morphology of molds isolated from fresh *A. sinensis* with postharvest disease during different stages of storage. (**A**) *Mucor hiemalis*; (**B**) *Actinomucor elegans*; (**C**) *Clonostachys rosea*; (**D**) *Penicillium polonicum*; (**E**) *Penicillium crustosum*; (**F**) *Aspergillus versicolor*; (**G**) *Alternaria alternata*; (**H**) *Trichoderma atroviride*; (**I**) *Fusarium solani*. (**A**–**C**, etc., left represents front, right represents back).

**Figure 3:**
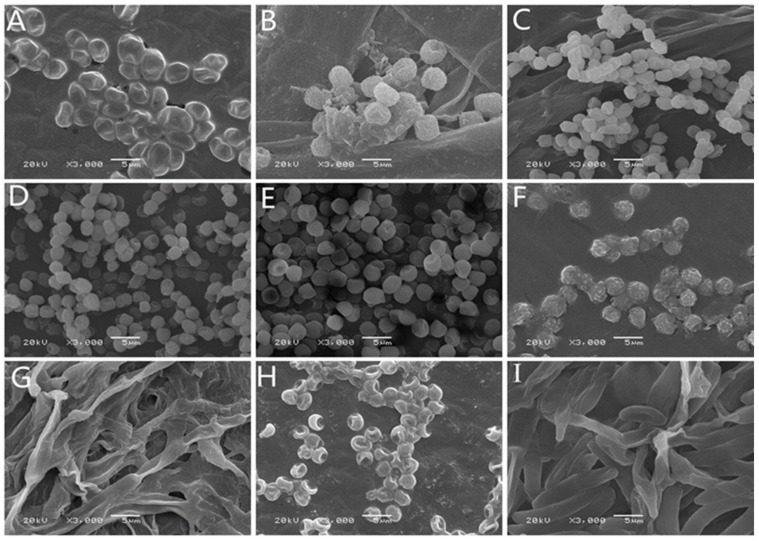
Morphology of spores from isolated from fresh *A. sinensis* with postharvest disease during different stages of storage. (**A**) *Mucor hiemalis*; (**B**) *Actinomucor elegans*; (**C**) *Clonostachys rosea*; (**D**) *Penicillium polonicum*; (**E**) *Penicillium crustosum*; (**F**) *Aspergillus versicolor*; (**G**) *Alternaria alternata*; (**H**) *Trichoderma atroviride*; (**I**) *Fusarium solani*.

**Figure 4:**
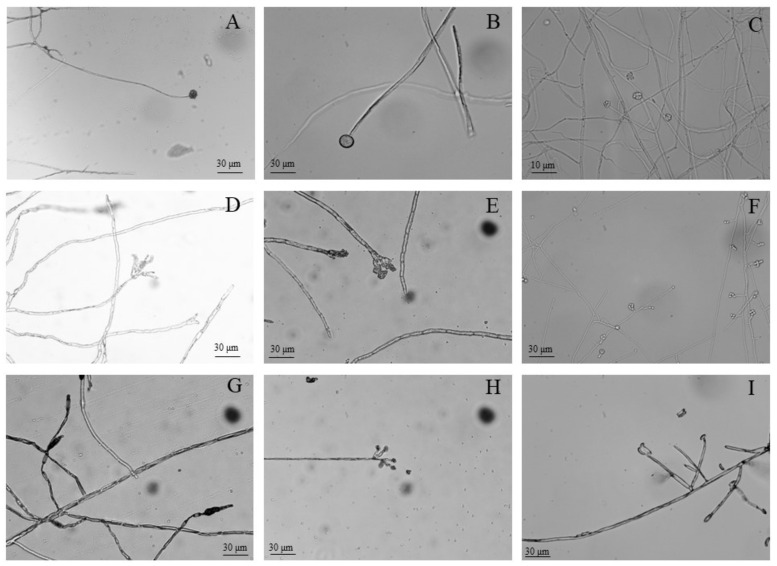
Morphology of conidiophores of molds isolated from fresh *A. sinensis* with postharvest disease during different stages of storage. (**A**) *Mucor hiemalis*; (**B**) *Actinomucor elegans*; (**C**) *Clonostachys rosea*; (**D**) *Penicillium polonicum*; (**E**) *Penicillium crustosum*; (**F**) *Aspergillus versicolor*; (**G**) *Alternaria alternata*; (**H**) *Trichoderma atroviride*; (**I**) *Fusarium solani*.

After isolation and purification, molds associated with various postharvest symptoms in *A. sinensis* during different storage periods were cultivated on PDA medium. Colony morphology (Figure 2), spore morphology (Figure 3), and conidiophore morphology (Figure 4) of the nine isolates were observed and described (Table 1).

Isolate 7–1 colonies resembled cotton wool, and were grey in color at the front, with beige at the back, irregularly outlined edges, and well-developed mycelia (Figure 2A). Isolate 7–1 spores were spherical and transparent (Figure 3A), with few symbiotic sporophore branches (Figure 4A). Isolate 7–2 also resembled cotton wool and was creamy white in color at the front and grey at the back, with an irregular colony outline (Figure 2B); spores were spherical (Figure 3B), and there was a larger sporangium at the tip of the main branch of the sporophore (Figure 4B). Isolate 14–1 colonies were fluffy in texture, and colored white at the front and light yellow at the back, with a regular outline and prostrate mycelia (Figure 2C); the spores were spherical or near spherical (Figure 3C), and the sporophore was erect and branched (Figure 4C). Isolate 21–1 formed concentric colonies that were dark green in color at the front, with a white radially wrinkled margin, and light yellow at the back (Figure 2D). The spores of isolate 21–1 were rosulate, and near-spherical (Figure 3D), with broom-shaped sporophores (Figure 4D). Isolate 21–2 formed densely textured colonies that were dark green at the front, with white irregular outlines at the edges, and light yellow at the back (Figure 2E); the spores were rosulate and near-spherical (Figure 3E), with short, broom-shaped, rough sporophores (Figure 4E). Isolate 21–3 formed concentric, velvety colonies, with green centers at the front, light yellow at the back, and white radial grooves at the edges (Figure 2F); the spores were spherical (Figure 3F), and the sporophores were expanded at the tip into a hemispherical sporangium (Figure 4F). Isolate 28–1 resembled cotton wool and was grey-brown at the front and dark brown at the back, with irregular margins and thick and loose mycelia (Figure 2G); the spores were stick-like, with tabula (Figure 3G), and the sporophores were solitary or in clusters, and mostly unbranched (Figure 4G). Isolate 28–2 was cottony, dark green at the front and light green at the back, with irregular margins (Figure 2H); the spores were subspherical or ovoid, with a depressed middle (Figure 3H), and the sporophores had short lateral branches, with small bottle-shaped pedicels formed at the end of the branches (Figure 4H). Isolate 49–1 was thin and fluffy, and light purple at the front, light pink at the back, with a white radiating edge and tall and dense mycelia (Figure 2I); the spores were ovate or ellipsoidal (Figure 3I), with branched or unbranched sporophores (Figure 4I).

### 2.4. Molecular Biology Identification of Isolates

**Figure 5:**
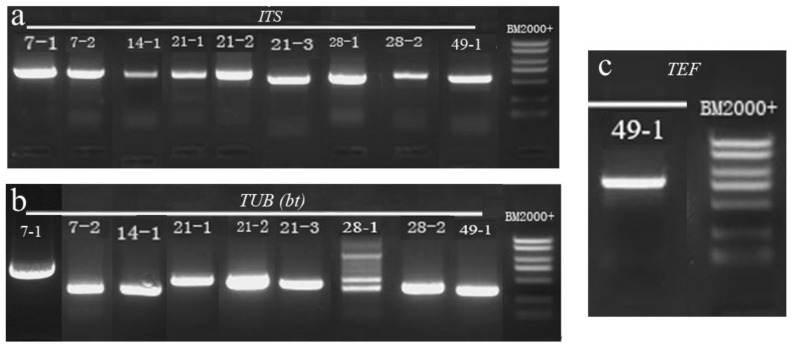
Gel electrophoresis images of PCR amplification products. (**a**) *ITS*; (**b**) *TUB*; (**c**) *TEF*.

**Figure 6:**
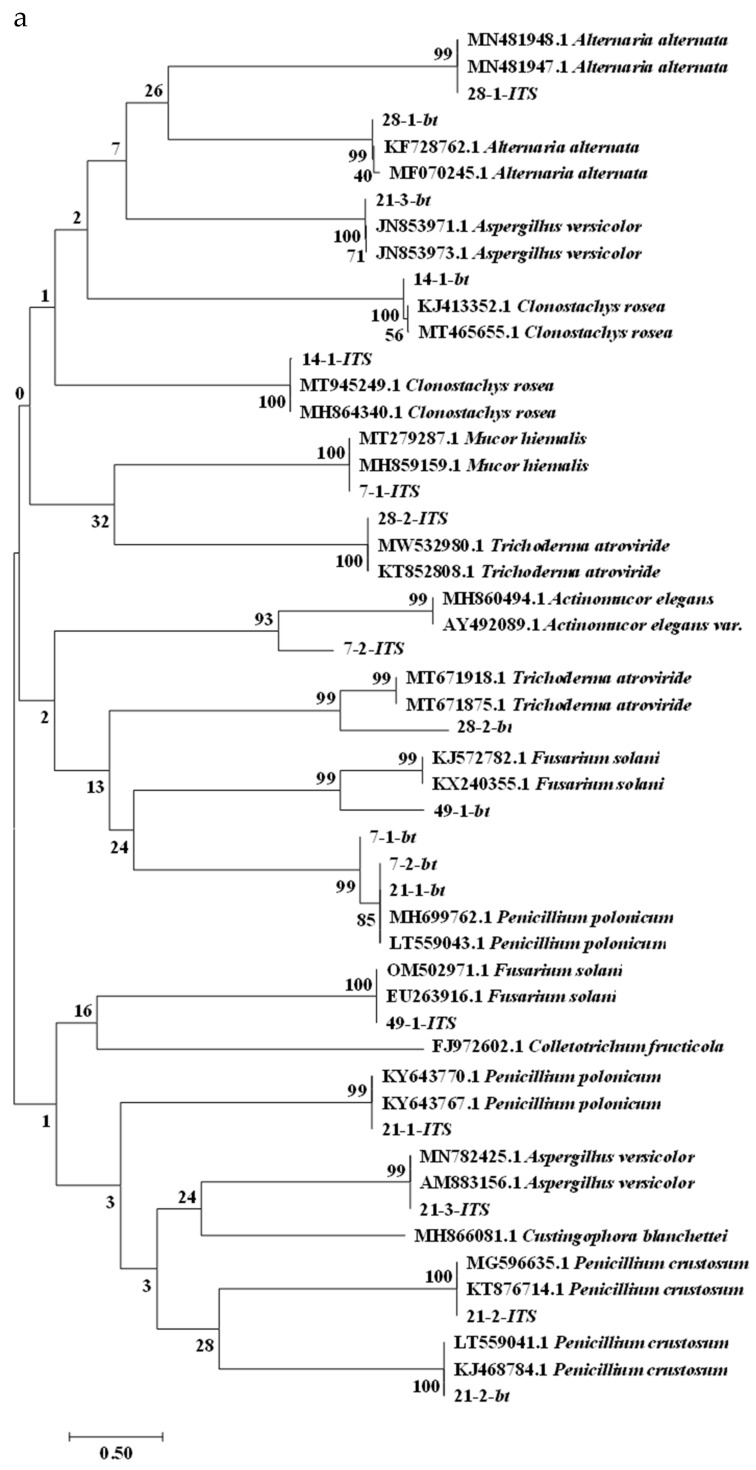

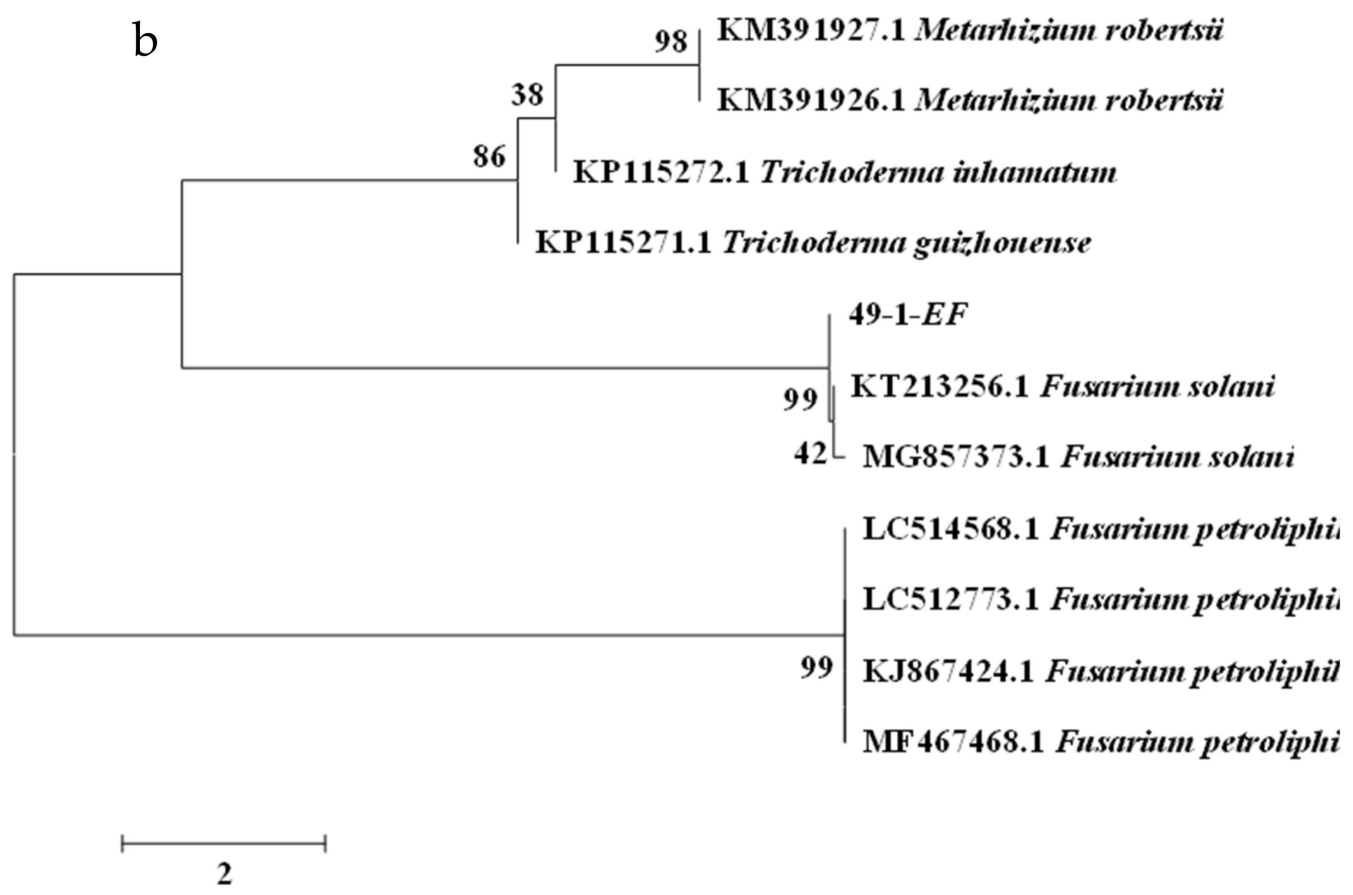
Phylogenetic trees based on analysis of different mold genes. (**a**) *ITS* and *TUB*; (**b**) *TEF*.

To further characterize the isolates that caused deterioration of freshly-harvested *A. sinensis* during different stages of storage, we subjected the nine isolates to molecular biological identification analysis. PCR amplification results indicated that the isolates had *ITS* sequences of 636 base pair (bp) for 7–1, 701 bp for 7–2, 597 bp for 14–1, 591 bp for 21–1, 557 bp for 21–2, 551 bp for 21–3, 552 bp for 28–1, 610 bp for 28–2, and 558 bp for 49–1 (Figure 5a). *TUB* sequences were 450 bp for 7–1, 453 bp for 7–2, 358 bp for 14–1, 456 bp for 21–1, 442 bp for 21–2, 394 bp for 21–3, 324 bp for 28–1, 348 bp for 28–2, and 327 bp for 49–1 (Figure 5b). For 49–1, the *TEF* sequence was 708 bp (Figure 5c). The PCR amplicons were sequenced and searched by BLAST against the NCBI database to identify homologous sequences. Phylogenetic trees of *ITS* and *TUB*, *TEF* sequences were constructed using MEGA7 software with the neighbor-joining method (Figure 6). Regarding the phylogenetic tree of *ITS* and *TUB* sequences (Figure 6a), the *ITS* sequences suggested that isolate 7–1 was on the same evolutionary branch as *Mucor hiemalis* (homology, 100%); isolate 7–2 was on the same evolutionary branch as *Actinomucor elegans* (homology, 93%); isolate 14–1 was on the same evolutionary branch as *Clonostachys rosea* (homology, 100%); isolate 21–1 was on the same evolutionary branch as *Penicillium polonicum* (homology, 99%); isolate 21–2 was on the same evolutionary branch as *Penicillium crustosum* (homology, 100%); isolate 21–3 was on the same evolutionary branch as *Aspergillus versicolor* (homology, 99%); isolate 28–1 was on the same evolutionary branch as *Alternaria alternata* (homology, 99%); isolate 28–2 was on the same evolutionary branch as *Trichoderma atroviride* (homology, 100%); and isolate 49–1 was on the same evolutionary branch as *Fusarium solani* (homology, 100%). The *TUB* sequences indicated that isolates 7–1 and 7–2 were both in the same evolutionary branch as *P. polonicum*, unlike the results of the *ITS* sequence; however, based on our molecular data together with the results of morphological characterization of isolates 7–1 and 7–2, we concluded that they were clearly not *Penicillium* species, but were *Mucormycete* species. The results of *TUB* sequences from isolates 14–1, 21–1, 21–2, 21–3, 28–1, 28–2, and 49–1 were all consistent with those of the *ITS* sequences. Therefore, combined with their morphology and biological characteristics, isolate 14–1 was identified as *C. rosea*, isolate 21–1 as *P. polonicum*, isolate 21–2 as *P. crustosum*, isolate 21–3 as *A. versicolor*, isolate 28–1 as *A. alternata*, isolate 28–2 as *T. atroviride*, and isolate 49–1 as *F. solani*. To further accurately characterize isolate 49–1, a separate *TEF* phylogenetic tree (Figure 6b) was generated, which indicated that isolate 49–1 was located in the same evolutionary branch as *F. solani* (homology, 99%), further supporting identification of isolate 49–1 as *F. solani*. The results of the phylogenetic trees are shown in Table 2.

Based on the observation of disease symptoms, and the results of morphological and molecular biological characterization, we identified nine isolates during different stages of storage of fresh *A. sinensis*. For the nine isolates, some isolates can cause serious postharvest disease. Nevertheless, other isolates were not established pathogens. Therefore, we performed pathogenicity tests for the nine isolates.

### 2.5. Pathogenicity Testing

**Figure 7:**
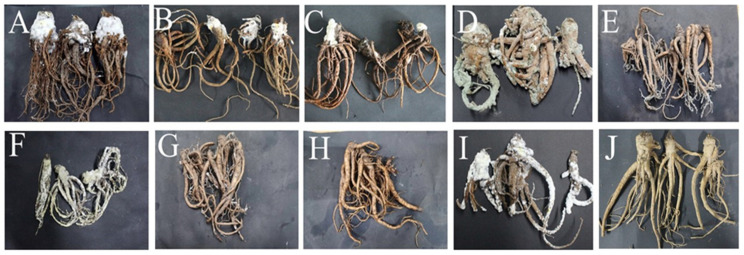
Pathogenicity testing of molds isolated from fresh *A. sinensis* with postharvest disease during different stages of storage. (**A**) *Mucor hiemalis*; (**B**) *Actinomucor elegans*; (**C**) *Clonostachys rosea*; (**D**) *Penicillium polonicum*; (**E**) *Penicillium crustosum*; (**F**) *Aspergillus versicolor*; (**G**) *Alternaria alternata*; (**H**) *Trichoderma atroviride*; (**I**) *Fusarium solani*; (**J**) Healthy *A. sinensis*.

Tests were conducted according to Koch’s postulates to assess the pathogenicity of the molds isolated during postharvest deterioration of *A. sinensis*. After 28 days of incubation, *A. sinensis* spray inoculated with isolate 7–1 (*M. hiemalis*) had grey-white mycelia and mold that had grown rapidly and covered the main root (Figure 7A). Inoculation with isolate 7–2 (*A. elegans*) resulted in rapid growth of white mycelia and expansion of colonies (Figure 7B). These two pathogens caused mucor rot of *A. sinensis*. Spraying with isolate 14–1 (*C. rosea*) resulted in expanding pink-white fluffy colonies, which gradually changed to yellow, causing pink rot of *A. sinensis* (Figure 7C). Inoculation with isolate 21–1 (*P. polonicum*) led to dark green colonies covering the surface of *A. sinensis*, with soft rot and mildew in tissue at the lesion (Figure 7D). Spraying with isolate 21–2 (*P. crustosum*) generated dark green colonies infesting the fibrous roots of *A. sinensis* (Figure 7E); hence, the two *Penicillium* species caused typical blue mold of *A. sinensis*. Inoculation with isolate 21–3 (*A. versicolor*) resulted in the rapid expansion of white colonies that covered the whole surface of *A. sinensis* and gradually turned yellow, indicating that the isolate caused aspergillosis of *A. sinensis* (Figure 7F). Spray inoculation with isolate 28–1 (*A. alternata*) resulted in grey mycelium infestation of the fibrous roots of *A. sinensis*, with expanding colonies and deepening color. However, there was no obvious disease (Figure 7G). Spraying with isolate 28–2 (*T. atroviride*) resulted in no visible disease symptoms, consistent with the condition of *A. sinensis* at the time of natural morbidity (Figure 7H). Inoculation with isolate 49–1 (*F. solani*) resulted in white fluffy colonies covering the whole *A. sinensis* sample, which gradually changed to light purple, and caused severe fusarium rot (Figure 7I). Control group *A. sinensis* showed no decay symptoms throughout the storage period (Figure 7J). Overall, *A. sinensis* spray inoculated with the nine pathogens developed various typical symptoms similar to the original natural symptoms. Further, the nine isolates were re-isolated, purified, and identified, and the resulting isolates had the same morphological and molecular biological characteristics.

### 2.6. Effect of Ozone Treatment on Postharvest Diseases of Fresh A. sinensis

**Figure 8:**
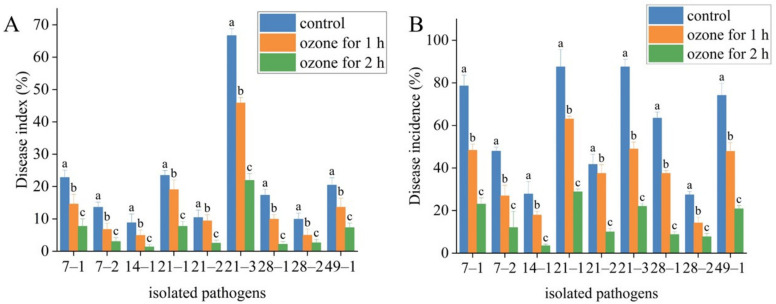
Effect of ozone treatment on disease index (**A**) and disease incidence (**B**) in fresh *A. sinensis* inoculated with 9 molds at 63 days of storage. The different letters indicate significant differences during the same storage period (*p* < 0.05).

The change in disease index and disease incidence showed that ozone treatment effectively suppressed the development of postharvest diseases in *A. sinensis* (Table 3). Moreover, the suppression effect of ozone treatment for 2 h was better than that for 1 h. For instance, *M. hiemalis* inoculation had disease index of 22.77% and disease incidence of 78.57% in control. Ozone treatment for 1 h had a disease index of 14.58% and disease incidence of 48.33%. Ozone treatment for 2 h had a disease index of 7.69% and disease incidence of 23.08%. *P. polonicum* inoculation had the disease index of 23.44% and the disease incidence of 87.5% in control. Ozone treatment for 1 h reduced the disease index and disease incidence to 19.02% and 63.04%, respectively, and ozone treatment for 2 h reduced the disease index and disease incidence to 7.69% and 28.85%, respectively. Similar results were observed in *A. sinensis* inoculated with other isolates (Figure 8).

### 2.7. Effect of Ozone Treatment on the Mycotoxin Accumulation in the Lesion Tissue of A. sinensis

**Figure 9:**
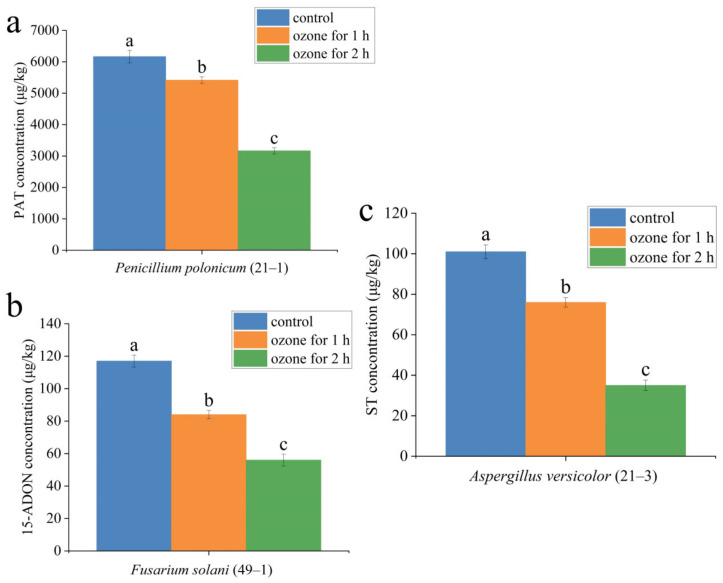
Effect of ozone treatment on the mycotoxin accumulation in the rotten tissue of fresh *A. sinensis* inoculated with *Penicillium polonicum* (21–1), *Fusarium solani* (49–1) and *Aspergillus versicolor* (21–3), (**a**) PAT concentration; (**b**) 15-ADON concentration; (**c**) ST concentration. The different letters indicate significant differences during the same storage period (*p* < 0.05).

Ozone treatment also significantly suppressed mycotoxin production in the lesion part of fresh *A. sinensis* with postharvest disease (Table 4). For instance, in the lesion part of *A. sinensis* infected by *P. polonicum* (Figure 9a), patulin (PAT) (6166 μg/kg) was detected in the control group. Ozone exposure for 1 h reduced PAT concentration to 5417 μg/kg, and the concentration of PAT was decreased to 3166 μg/kg after 2 h of ozone exposure (Figure 9a). Similarly, in the lesion part of *A. sinensis* fusarium rot caused by *F. solani*, 15-acetyl-deoxynivalenol (15-ADON) (117 μg/kg) was detected in the control group, 15-ADON (84 μg/kg) was detected after 1 h of ozone exposure, and 15-ADON (56 μg/kg) was detected after 2 h of ozone exposure *A. sinensis*. The same change trend was observed in the lesion part of *A. sinensis* aspergillosis caused by *A. versicolor* (Figure 9c). Aflatoxin B1, ochratoxin A, and T-2 toxin were not detected in fresh *A. sinensis* infected by other pathogens.

## 3. Discussion

To our knowledge, this is the first report on the postharvest disease of fresh *A. sinensis* and the corresponding pathogenic molds during different stages of storage after harvesting, as well as analysis of the effect of ozone treatment on the inhibition of post-harvest disease and mycotoxin accumulation in diseased tissues of *A. sinensis*. Postharvest disease becomes more serious with extended storage time. A total of nine molds were isolated from the diseased tissues of fresh *A. sinensis* with postharvest disease during different stages of storage and characterized based on their morphological and molecular biological features. Additionally, ozone exposure significantly decreased the development of postharvest disease and mycotoxin production in fresh *A. sinensis* infected by the nine isolates.

The earliest disease observed was mucor rot caused by molds of the genus *Mucor* during postharvest storage of *A. sinensis*, including *M. hiemalis* and *A. elegans*. Yazdi et al. [16] isolated and identified *M. hiemalis* from the soil, and described similar spores and conidiophore morphology to those observed in this study, where the spores were spherical and hyaline, and the conidiophore had few symbiotic branches. He et al. [17] isolated *A. elegans* from Sichuan Taihe Mucor Douchi, and reported that it had white colonies at the beginning, then changed to pale yellow later, with erect and branched sporangiophores, and spherical sporangia, which was similar to the morphology of the spores of the pathogen isolated from mucor rot of *A. sinensis* in this study. *Mucor* grows very rapidly, particularly at temperatures of 18–30 °C, with relatively high humidity. *Mucor* spores are widely distributed in the air, soil, and plant residues, can spread on air currents [18], and are the main source of infection for primary infestations. *A. sinensis* is harvested annually at the end of October, when local temperatures are 20–30 °C during the day and 15 °C–20 °C at night, which are favorable for *Mucor* infection and host colonization, with fast growth speed. Thus, the first postharvest disease observed was mucor rot.

*C. rosea*, which caused pink rot of *A. sinensis*, was isolated and characterized during the 14th day of storage. Zhang et al. [19] isolated *C. rosea* from litchi, and found that it had white snowy fluffy colonies, spherical or elliptical spores, and broom-shaped conidiophores, which is generally consistent with our observations. *C. rosea* has been used as a biocontrol agent against spot blotch in barley [20] and corn stalk rot [21]. However, it can also cause barley root rot [22]. During the 21st day of storage, typical and severe postharvest blue mold was observed, caused by *P. polonicum* and *P. crustosum*. *P. polonicum* was more pathogenic in *A. sinensis*, as large numbers of spores covering the surface of *A. sinensis* were observed, whereas *P. crustosum* infections were limited to the lateral root of *A. sinensis*. Shanawaer et al. [23] isolated *P. polonicum* from mold rot of Jujube fruit, and reported that it formed dark green colonies, with mostly spherical spores, and branched broom-shaped conidiophores, largely consistent with our observations of morphological characteristics. *P. polonicum* can metabolize and generate patulin. In the present study, patulin (PAT) was detected in *A. sinensis* infected by *P. polonicum*. PAT is a secondary metabolite produced by molds, including *Aspergillus* and *Penicillium*, under favorable conditions, has genotoxic, teratogenic, mutagenic, and immunosuppressive effects [24]; and is a common contaminant of pome fruits and their corresponding products. The European Union set maximum limits of PAT in fruit juice of 50 μg/kg and in infant food of 10 μg/kg [25]. However, there are no standard PAT level limits in Chinese herbal medicines. Chen et al. [26] isolated *P. crustosum* from an experimental field and laying hen farms at Anhui Academy of Agriculture, and reported that it had green colony centers, septate hyphae, and short conidiophores, similar to the morphological characteristics observed in this study. Moreover, *P. crustosum* has been reported to exhibit weak pathogenicity and could be useful as a biocontrol pathogen to inhibit the growth of *Colletotrichum capsici* and *Phytophthora capsici*. *Penicillium* species were reported to be “wound pathogens” [27], which mainly invade host plants via wounds or lesions, due to fruit cracking or mechanical damage during harvesting, transport, handling, and storage. Therefore, to prevent infection of *A. sinensis* by *Penicillium* species, care should be recommended during packing house handling.

The most serious postharvest disease detected was aspergillosis caused by *A. versicolor*. As shown by pathogenicity testing, *A. versicolor* spores covered the whole *A. sinensis* tissue. *Aspergillus* spp. have been reported among the dominant molds contaminating Chinese herbal medicines [28]. Zhang et al. [29] isolated *A. versicolor* from *Annamocarya sinensis* tissues and found that it had dark green colonies with white margins, a velvety texture, central depression, yellow-brown downy projections, and spherical spores, consistent with the morphological characteristics observed in this study. More importantly, *A. versicolor* metabolizes and produces sterigmatocystin (ST). ST exhibits immunotoxicity and strong carcinogenicity [30]. ST was detected in *Ilex asprella* [31], and Zheng et al. [32] demonstrated that ST was the most common mycotoxin in the traditional Chinese herbs they analyzed. The California Department of Health Services used TD50 values from the Cancer Potency Database to determine a “no significant risk” intake level for ST of 8 μg/kg body weight/per day (for a 70 kg adult) [33].

During the 28th day of storage, *A. alternata* and *T. atroviride* were identified. Gao et al. [34] obtained *A. alternata* from *Astragalus membranaceus* root rot in Shanxi Province, and reported that it had rounded colonies with neat edges, flocculent mycelia, and were initially white, then turning grey-green, dark green to dark green-brown, and grey-black. Most spores were inverted mallet-shaped, or inverted pear-shaped, and a few were elliptical. *A. alternata* is the main pathogen causing pomegranate fruit rot during storage [35] and is also a “latent infection pathogen” that remains in the host when the plants grow in the field until optimal growth conditions (such as high temperature, high humidity, or plant harvest) occur [27]. In this study, *A. alternata* was not pathogenic for *A. sinensis*, as no obvious disease symptoms were detected during pathogenicity testing. *T. atroviride* was another isolate identified on the 28th day of storage. Wu et al. [36] obtained *T. atroviride* from *Artemisia annua*, with colonies that were white at the front and colorless at the back. Conidiogenous cells were initially white, gradually turning yellow-green, and mature conidiogenous cells were dark green. Spores were unicellular, subspherical, or ovoid, and 3.0–5.0 μm in size, generally consistent with the morphological features observed in our study. Pathogenicity testing indicated that *T. atroviride* did not cause visible postharvest disease. Indeed, *T. atroviride* is often applied as a biocontrol agent to suppress pathogen growth and can also have growth-promoting effects on many plants, including ryegrass [37] and *Trifolium repens* [38].

During the 49th day of storage, *F. solani* was isolated from *A. sinensis* root rot. *Fusarium* species are common contaminants of Chinese herbal medicines, but most have been identified in the field. Li et al. [39] obtained *F. solani* from *Cymbidium hybrid* root rot tissues, and found that it generated thin, downy, light purple colonies, with earthy central colonies on the reverse side, and ovoid or elliptical spores, in general agreement with the results of this study. *F. solani* can metabolize and produce trichothecene, 15-ADON, as one kind of type B trichothecene, was detected in *A. sinensis* fusarium rot lesions caused by *F. solani*. 15-ADON has various toxic effects, including anorexia, growth retardation, immunotoxicity, and impaired reproduction [40], posing a serious threat to human health. There are previous reports of 15-ADON contamination in *Fusarium* species-infested wheat spikes [41] and maize [42], but no report in Chinese herbs. No maximum allowable legal limits of 15-ADON have yet been set [42]. *F. solani* is the main pathogen that causes root rot of carrots [43] and celery [44]. *Fusarium* has also been referred to as a “latent infection pathogen” [45], which can infect plants in the growing stage (in the field), then begin to develop and colonize further when optimal growth conditions occur or the plants are harvested.

A better understanding of the occurrence of diseases during postharvest storage of *A. sinensis* can help to develop and design *A. sinensis* preservation and control strategies. However, there were very few studies on the ozone control of postharvest diseases and mycotoxin accumulation in *A. sinensis*. Ozone treatment is considered a cost-effective and eco-friendly food processing technology to preserve the fruit quality. A study by Gao et al. [46] showed that ozone exposure reduced the number of molds on the grape surface, destroyed the colony structure, and controlled the occurrence of diseases. Chen et al. [47] confirmed that ozone fumigation at the dose of 15.008 mg m^−3^ maintained the postharvest quality of cantaloupe. Zhang et al. [48] showed that ozone treatment reduced the incidence of fruit decay and maintained the firmness of winter jujube fruit. Xue et al. [49] showed that 1.10 mg/L ozone treatment for 120 min more significantly controlled muskmelon fusarium rot development than 60 min ozone exposure. In the present study, ozone treatment not only significantly suppressed the postharvest disease of the infected *A. sinensis,* but there was also an ozone-treatment-time-dependent manner. For instance, compared with the control group, ozone treatment for 1 h reduced the disease index and disease incidence by 35.97 and 38.49%, respectively, in *A. sinensis* infected by *M. hiemalis*. Moreover, ozone application for 2 h reduced the disease index and disease incidence by 66.23% and 70.62%, respectively. Similarly, *A. sinensis* infected by *C. rosea* showed the better inhibitory effect after 2 h of ozone treatment than 1 h of ozone treatment. There were two reasons for this. On the one hand, ozone can directly inhibit the mycelial growth and spore germination of pathogens [50]. On the other hand, ozone inhibition is related to the mode of ozone treatment. Similar results were obtained by Guo et al. [51] when they studied the effect of ozone on controlling gray mold in grapes. Ozone could concentration- and time-dependently damage the integrity of the plasma membrane of *Botrytis cinerea* and reduce conidia germination. The higher the ozone concentration and the longer the treatment time, the better the effect.

Mycotoxins are secondary metabolites of pathogenic molds produced under favorable conditions, having carcinogenic, teratogenic, or mutagenic effects on human health [40]. The results in the present study showed that 2 mg L^−1^ ozone treatment significantly reduced the mycotoxin accumulation in diseased *A. sinensis* tissues, and 2 h ozone exposure was more effective than the 1 h ozone exposure. The reason for this maybe that ozone treatment directly acted and killed the pathogens by destroying the cell structure of the pathogen. Similar results were confirmed by Wang et al. [52], who reported a 39.16% and 53.48% reduction in DON level after 60- and 90-min exposure to 75 mg/L gaseous ozone, respectively. Additionally, ozone can also directly react with the structure of mycotoxin, leading to mycotoxin degradation. For instance, Xue et al. [49] suggested that the mechanism of ozone treatment is to directly act on the chemical structure of NEO, thereby destroying the structure of NEO.

## 4. Conclusions

In summary, a total of nine mold species were isolated and identified from postharvest *A. sinensis* tissues showing signs of disease during different stages of storage in this study. The nine molds had various colony, spore, and conidiophore morphologies and seven caused different typical symptomatic postharvest diseases. Disease occurrence during postharvest storage of *A. sinensis* not only leads to economic losses. More importantly, some pathogenic molds produced mycotoxins (PAT, 15-ADON, ST) which pose a threat to human health. Ozone treatment effectively inhibited the development of disease and mycotoxin accumulation of *A. sinensis*. Therefore, to accurately and efficiently control postharvest disease during storage of *A. sinensis*, diverse management strategies should be precisely targeted toward different pathogenic molds. For some types of pathogens, such as “latent infection pathogens”, control should be carried out in the field, while for “wound infection pathogens”, management of harvesting processes and the postharvest storage environment should be strengthened. In addition, the mechanism of ozone control of postharvest disease and mycotoxin production should be further studied, to provide a basis for postharvest disease control and quality improvement of *A. sinensis*.

## 5. Materials and Methods

### 5.1. Isolation of Mold Pathogens

Fresh *A. sinensis* (cv. Mingui 2, A variety of *Angelica sinensis* selected from the Arid Agriculture Centre in Dingxi, Gansu Province) was harvested from the Chinese herb medicine planting base in Min County (location: 35° N, 104° E), Gansu Province. Similar size samples of fresh *A. sinensis*, without visible mechanical damage or insect pests, were selected and transferred to the Chemical Biological Laboratory at Gansu Agricultural University, then placed in sterile preservation bags, and stored in darkness for 7, 14, 21, 28, 35, 49, and 63 days (20 °C, 50% RH). The naturally occurring symptoms were carefully observed during storage. Groups included 20 samples of *A. sinensis* and there were three replicates for each group; hence, a total of 420 samples were included in the entire experiment (20 samples × 3 replicates × 7 time points).

Fresh *A. sinensis* tissues with typical disease symptoms during different storage stages were selected, and fragments (3 × 3 mm in size) were collected from healthy-diseased tissue junctions with a sterile blade, then surface disinfected by immersing them in 1% NaClO for 3 min, and washed with sterile water three times. Disinfected *A. sinensis* fragments were inoculated on potato dextrose agar (PDA) medium and cultured in an incubator at 28 °C for 3–5 days. Then, the mold colonies obtained were grouped according to colony morphology, and representative colonies of specific groups randomly selected and isolated by streaking on PDA plates until a single mold colony was obtained. Subsequently, individual colonies were incubated on PDA plates at 25 °C. After 4–5 cycles of isolation and purification, a single purified colony was harvested.

### 5.2. Identification of Molds Potentially Causing Postharvest Disease during Different Storage Stages of Fresh A. sinensis

#### 5.2.1. Morphological Identification of Isolated Molds

Morphological characterization is an important and indispensable method for fungus identification, and mainly involves observation of colony morphology and macro and microconidia characteristics [53]. Spore suspensions (1 × 10^6^ spores/mL) of purified isolates were inoculated onto PDA medium and incubated at 25 °C for 7 days. Subsequently, colony morphology and pigment secretion were observed and described. Spore morphology was observed under a scanning electron microscope (JEOL JSM-5910LV, Tokyo, Japan) and conidiophore morphology was observed by laser confocal microscopy (FV3000, Japan, Tokyo).

#### 5.2.2. Molecular Biological Identification of Isolated Molds

##### Genomic DNA Extraction

Spore suspensions (1 × 10^6^ spores/mL) of isolates were inoculated in 100 mL of potato dextrose broth medium and incubated at 25 °C for 7 days. Then, mycelia were collected after filtering and ground into a fine powder under liquid nitrogen. UNlQ-10 column mold genomic DNA extraction kits (Sangon, Shanghai, China) were used to extract DNA from the obtained mycelia, according to the manufacturer’s instructions.

##### PCR Amplification and Sequencing

Internal transcribed spacer (*ITS*) and β-tubulin (*TUB*) are conserved sequences in mold genes that are often used for molecular identification of mold species. Translation elongation factor (*TEF*) is a specific primer that is often used for identification of *Fusarium* species. Extracted mold DNA was used as a template for PCR amplification with the primers *ITS1* (5′-TCCGTAGGTGAACCTGCGG-3′) and *ITS4* (5′-TCCTCCGCTTATTGATATGC-3′) for nine isolates; *bt2a* (5′-GGTAACCAAATCGGTGCTGCTTTC-3′) and *bt2b* (5′-ACCCTCAGTGTAGTGACCCTTGGC-3′) for nine isolates; and *EF1* (5′-ATGGGTAAGGA(A/G)GACAAGAC-3′) and *EF2* (5′-GGA(G/A)GTACCAGT(G/C)ATCATGTT-3′) for *Fusarium* spp. PCR amplification reaction systems (50 μL) contained: 46–47 μL 1× Taq PCR Mix, including 1 μL of each upstream and downstream primers, and 1 μL of template DNA. The PCR amplification procedure was as follows: first, pre-denaturation at 94 °C for 5 min; then, denaturation at 94 °C for 10 s, followed by annealing at 53 °C for 10 s and extension at 72 °C for 30 s, 30 cycles, and a final extension at 72 °C for 5 min. Obtained amplification products were separated by electrophoresis on 2% agarose gel. Amplified fragments were sequenced by China Beijing Bomede Biotechnology Co., Ltd. and the sequencing results were subjected to homology analysis using NCBI BLAST (https://www.ncbi.nlm.nih.gov/, accessed on 9 July 2022), with appropriate sequences selected to construct phylogenetic trees using the neighbor-joining method in MEGA7 software (Molecular Evolutionary Genomics Analysis Version 7). Molecular biological analysis was combined with morphological characterization to identify mold species.

### 5.3. Pathogenicity Testing of Isolates

Fresh *A. sinensis* was harvested from an open field, and plants without obvious damage or disease were selected, then disinfected with 0.1% NaClO for 15 min, washed with distilled water three times, and air dried naturally at room temperature. Spore suspensions (1 × 10^6^ spores/mL) of molds were inoculated by spraying on the surface of disinfected *A. sinensis*. Then, the inoculated *A. sinensis* was placed in sterile sealed bags and stored at room temperature for 28 days (20 °C, 50% RH). *A. sinensis* with sterile water sprayed on the surface served as a control group. Disease symptoms were recorded and described [54]. Molds were re-isolated and identified, and it was observed whether they had similar morphological characteristics as the original isolates determined. Isolates that conformed to Koch’s postulates were selected for further study.

### 5.4. Effect of Ozone Treatment on Postharvest Disease of Fresh A. sinensis

Healthy *A. sinensis* were disinfected by immersion in 0.1% NaClO for 15 min, and then rinsed with sterile water three times. Spore suspensions (1 × 10^6^ spores/mL) of isolated pathogens were prepared and sprayed onto the surfaces of disinfected fresh healthy *A. sinensis*. After inoculation, *A. sinensis* were placed in airtight transparent bags (80 cm long × 60 cm wide) (25 °C and 75% relative humidity) and ozone fumigation treated for 1 h and 2 h daily, over 7 days. Gaseous ozone was generated by OSAN ozone generator (Aoshan Huanbao Technology Industry Co. Ltd., Dalian, China), and the concentration of 2 mg L^−1^ was adjusted according to our previous publication [55]. No ozone treatment was used as control. Subsequently, the treated tissues were stored in plastic bags (22 ± 2 °C, 75–80%) for 63 d. Each treatment contained three replicates, and one replicate included 20 samples. Then, the disease index and disease incidence were measured and calculated according to Formulas (1) and (2) [56,57], as follows:

Disease Index = [sum (class frequency × score of rating class)/(Total number of plants × maximal disease index)] × 100(1)

Disease incidence = (Number of the infected plants/the number of plants sampled) × 100(2)

For the two formulae, disease level was classified into five categories: 0, 1, 2, 3, and 4 (Table 5) according to Cao et al. [58], with minor modifications.

### 5.5. Effect of Ozone Treatment on the Mycotoxin Accumulation in the Lesion Tissue of A. sinensis

The samples from lesion tissue in ozone-treated *A. sinensis* were collected and stored at −80 °C for mycotoxin analysis. Frozen samples (5.0 g) were ground in liquid nitrogen, then transferred to a 50 mL clean, dry centrifuge tubes, and the extraction solvent was added to extract mycotoxin. Various purification and detection methods were employed for different types of mycotoxin: patulin (PAT) [59], trichothecenes [60] and sterigmatocystin (ST) [61], aflatoxin B1 [62], ochratoxin A [62], and T-2 toxin [62] detection were respectively used according to the previously described methods.

### 5.6. Statistical Analysis

Disease index, disease incidence, and mycotoxin accumulation data were expressed as mean (± standard error) and analyzed with one-way analysis of variance (ANOVA). Statistical analyses were carried out using SPSS v. 17.0 (SPSS, Inc., Chicago, IL, USA), and differences were considered statistically significant at *p* < 0.05. The normality of data fitted to standard normal distribution of the equation: Y = (X−µ)/σ~N (0, 1). In addition, the data was obtained from the results of at least three parallel experiments, and the average mean and standard deviation (STDEV) were calculated using Excel 2016.

## Figures and Tables

**Table 1:** Morphological characteristics of pathogens isolated during different storage periods.

Colony Morphology					Microscopic Morphology	
Strain Number	Front Color	Back Color	Texture	Margin	Conidium	Conidiophore
7–1	grey	beige	cotton wool-like	irregular white	spherical, transparent	few symbiotic branches
7–2	creamy white	grey	cotton wool-like	irregular white	spherical	sporangium
14–1	white	light yellow	fluffy	regular white	spherical or near spherical	erect and branch
21–1	dark green	light yellow	concentric colony	white radial wrinkles	rosette-like bunches, nearly spherical	broom
21–2	dark green	light yellow	dense felt	irregular white	rosette-like bunches, near-spherical	broom
21–3	center into green	light yellow	concentric and velvety colony	white radial grooves	spherical	sporangium
28–1	brown	grey-brown	cotton wool-like	irregular black	stick-like	mostly unbranched
28–2	dark green	light green	cottony	irregular green	subspherical or ovoid	with short lateral branches
49–1	light purple	light pink	thin fluffy	white radial	ovate or elliptical	branched or unbranched

**Table 2:** *ITS*, *TUB*, and *TEF* sequences identification results.

A. *ITS* Sequence Identification Results		
Strain Number	Evolutionary Branch	Homology
7–1	*Mucor hiemalis*	100%
7–2	*Actinomucor elegans*	93%
14–1	*Clonostachys rosea*	100%
21–1	*Penicillium polonicum*	99%
21–2	*Penicillium crustosum*	100%
21–3	*Aspergillus versicolor*	99%
28–1	*Alternaria alternata*	99%
28–2	*Trichoderma atroviride*	100%
49–1	*Fusarium solani*	100%
**B. *TUB* sequence identification results**		
**Strain Number**	**Evolutionary Branch**	**Homology**
7–1	*Penicillium polonicum*	99%
7–2	*Penicillium polonicum*	85%
14–1	*Clonostachys rosea*	100%
21–1	*Penicillium polonicum*	85%
21–2	*Penicillium crustosum*	100%
21–3	*Aspergillus versicolor*	100%
28–1	*Alternaria alternata*	99%
28–2	*Trichoderma atroviride*	99%
49–1	*Fusarium solani*	99%
**C. *TEF* sequence identification results**		
**Strain Number**	**Evolutionary Branch**	**Homology**
49–1	*Fusarium solani*	99%

**Table 3:** Effect of ozone treatment on disease index and disease incidence in fresh *A. sinensis* inoculated with 9 molds at 63 days of storage.

Strain Number	Disease Index			Disease Incidence		
	Control	Ozone for 1 h	Ozone for 2 h	Control	Ozone for 1 h	Ozone for 2 h
7–1	22.77 ± 2.3	14.58 ± 3	7.69 ± 2.3	78.57 ± 5.1	48.33 ± 2.9	23.08 ± 2.9
7–2	13.59 ± 1.6	6.73 ± 1.9	3.0 ± 1.2	47.92 ± 1.9	26.92 ± 5	12.0 ± 7.6
14–1	8.8 ± 2.7	4.91 ± 1.6	1.34 ± 0.7	27.78 ± 5.8	17.86 ± 1.8	3.45 ± 0.8
21–1	23.44 ± 1.5	19.02 ± 3	7.69 ± 1.5	87.5 ± 8.1	63.04 ± 1.3	28.85 ± 1.3
21–2	10.42 ± 2.3	9.38 ± 1.9	2.5 ± 0.9	41.67 ± 4.8	37.5 ± 4.1	10.0 ± 1.6
21–3	66.67 ± 2.1	45.83 ± 1.7	21.88 ± 2.1	87.5 ± 3.6	48.89 ± 3.3	22.0 ± 1.3
28–1	17.31 ± 1.8	9.9 ± 1.4	2.17 ± 0.8	63.46 ± 2.8	37.5 ± 1.3	8.7 ± 1.3
28–2	9.89 ± 1.9	4.92 ± 1.9	2.6 ± 1.04	27.39 ± 1.6	14.17 ± 2.7	7.69 ± 1.5
49–1	20.42 ± 2.3	13.59 ± 2.8	7.29 ± 1.4	74.14 ± 5.7	47.83 ± 4.1	20.83 ± 1.5

**Table 4:** Effect of ozone treatment on the mycotoxin accumulation in the rotten tissue of fresh *A. sinensis* inoculated with *Penicillium polonicum*, *Fusarium solani* and *Aspergillus versicolor*.

Molds Species	Accumulation of Mycotoxins in Fresh *A. sinensis* Rotting Tissue								
	PAT (μg/kg)			15-ADON (μg/kg)			ST (μg/kg)		
	Control	Ozone for 1 h	Ozone for 2 h	Control	Ozone for 1 h	Ozone for 2 h	Control	Ozone for 1 h	Ozone for 2 h
*P. polonicum*	6166 ± 198.7	5417 ± 106.4	3166 ± 97.3	nd			nd		
*F. solani*	nd			117 ± 3.7	84 ± 2.6	56 ± 3.6	nd		
*A. versicolor*	nd			nd			101 ± 3.4	76 ± 2.3	35 ± 2.6

nd: not detected.

**Table 5:** Disease classification standard.

Disease Rating	Symptom
0	No disease
1	Fibrous root disease area 0~25% or primary root disease area 0~9%
2	Fibrous root disease area 25~50% or primary root disease area 10~25%
3	Fibrous root disease area is greater than 50% or the primary root disease area is 25~50%
4	Primary root disease area greater than 50%

## Data Availability

The data presented in this study are included in the article; further inquiries can be directed to the corresponding author.
